# Soil and foliar application of rock dust as natural control agent for two-spotted spider mites on tomato plants

**DOI:** 10.1038/s41598-020-69060-5

**Published:** 2020-07-21

**Authors:** Nicoletta Faraone, Rodger Evans, Julia LeBlanc, Neil Kirk Hillier

**Affiliations:** 1 Chemistry Department, Acadia University, Wolfville, NS B4P 2R6 Canada; 2 Biology Department, Acadia University, Wolfville, NS B4P 2R6 Canada

**Keywords:** Chemical ecology, Plant sciences, Entomology

## Abstract

Mineral-based products represent a valid alternative to synthetic pesticides in integrated pest management. We investigated the effects of a novel granite dust product as an agent for controlling two-spotted spider mites, *Tetranychus urticae* Koch (Acari: Tetranychidae), on tomato plants (*Solanum lycopersicum* L.). Two-choice tests for repellency and repulsiveness, and no-choice bioassays with different type of applications (soil, foliar, and soil–foliar) were used in order to evaluate performance and action of the product. Evaluation of epidermal micromorphology and mesophyll structure of treated plants and elemental analyses of leaves were performed. In repulsiveness experiments, almost all dust treatments significantly inhibited mites from migrating to and/or settling on the treated leaf. In repellency experiments, foliar and soil dust treatments were not significantly different from control. Significant mortality was observed for all dust treatments in two-choice and in no-choice bioassays, suggesting mites are susceptible to rock dust by contact, and by indirect interaction through the feeding on plants subjected to soil application of rock dust. Leaf epidermal micromorphology and mesophyll structure of treated plants showed structural variation due to mineral accumulation, which was also confirmed by elemental analyses of leaves. These results demonstrate for the first time that granite rock dust interacts with two-spotted spider mites by modifying pest behavior and via acaricidal action, providing more insights in understanding the mechanism of this novel natural product as pest management tool.

In the effort to find better alternatives to conventional synthetic pesticides and embrace reduce-risk strategy approaches in crop protection, mineral rock dusts offer an interesting option as natural products to prevent or control pests, diseases, and other plant pathogens, with a minimal impact on human health and the environment^1^. Deployed as foliar spray application or as soil amendment, mineral- and dust-based products (such as diatomaceous earth) have been widely used in crop protection, with increasing interest in the agricultural sector as crop pest management tools^2–5^.

Rock dust is a valuable source of silicon (in the form of amorphous silica, SiO_2_), which is one of the most abundant elements in the earth’s crust. It has beneficial effects for plants under a range of abiotic and biotic stresses^6–8^. Application of silicon in crops as a pre-harvest treatment provides a viable component of integrated management of insect pests and diseases as it does not leave pesticide residues in food or the environment, and it can be easily integrated with other pest management practices, including biological control^9,10^. When a powdered siliceous mineral is shaken with water, particles from large colloids to simpler ions can be dissolved in the aqueous solution forming different complex (such as colloidal silicic and orthosilicic acids)^11,12^. Silicon is absorbed by plant roots from the soil as monosilicic acid [(Si(OH)_4_], transported throughout the plant tissue via transpiration and deposited in plant epidermal cell walls^13^. Several studies have shown a role for silicon-based products in enhancing resistance of plants to insect herbivores including folivores^14,15^ and phloem feeders^16^, typically at a plant–herbivore trophic level. The protective effect of silica to plants against herbivores is related to the level of its accumulation and polymerization in plant tissues, forming a mechanical barrier that increases resistance to pest attack^17–19^.

Granite dust examined in the current study suggests similar effects and mode of action reported for other silica dust products, and it exerts repellent, insecticidal and anti-ovipositional activities against Lepidoptera^15^. In terms of the mode of action, it remains unclear precisely how granite dust affects herbivores leading to a reduction in insect performance and plant damage. In this context, we want to evaluate: (a) the effect of granite dust in controlling cell disrupting herbivores; (b) potential pest control differences within the application via the roots (as soil amendment) and/or as a leaf surface (foliar) application; (c) the defence mechanism involved (mode of action).

Two-spotted spider mite (TSSM), *Tetranychus urticae* Koch (Acari: Tetranychidae), was selected for study as a generalist pest which feeds on hundreds of plant species, with acceptance according to the nutritive and toxic constituents of the plant^20,21^. Because of its high rate of fecundity and short developmental time, management of TSSM is based on frequently rotating acaricides that have increased the development of resistant population^22,23^. As an alternative, biological control of TSSM in greenhouse conditions is commonly used with the employment of phytoseiid predatory mites^24^. Although effective in controlling TSSM population, they are highly sensitive to acaricides and fungicides, and unable to survive in temperate climates^25^. As host for our study, we selected tomato plants (*Solanum lycopersicum* L.) as representative crop for main greenhouse vegetable industry in Canada significantly affected by TSSM, and because the exposure to rock dust in preliminary studies induced changes on plant fitness and fruit quality (personal observation).

## Results

The elemental analysis performed on samples of rock dust demonstrated the high content of silicon present in the form of SiO_2_ (around 60% of the overall composition) (Table 1). The application of rock dust material via foliar and/or soil application resulted in acaricidal and repellent activity towards TSSM.

**Table 1:** Analyte mass (wt% abundance ± SE) of major and trace elements in granite rock dust samples (N = 11) detected by XRF recorded as oxides.

Element	Analyte mass
	wt% abundance
SiO_2_	62.33 ± 0.98
Al_2_O_2_	15.11 ± 0.27
Fe_2_O_3_	5.74 ± 0.42
CaO	5.76 ± 0.44
MgO	3.63 ± 0.40
Na_2_O	3.46 ± 0.13
K_2_O	2.10 ± 0.17
MnO	0.09 ± 0.01
TiO_2_	0.54 ± 0.06
P_2_O_5_	0.14 ± 0.02

**Figure 1:**
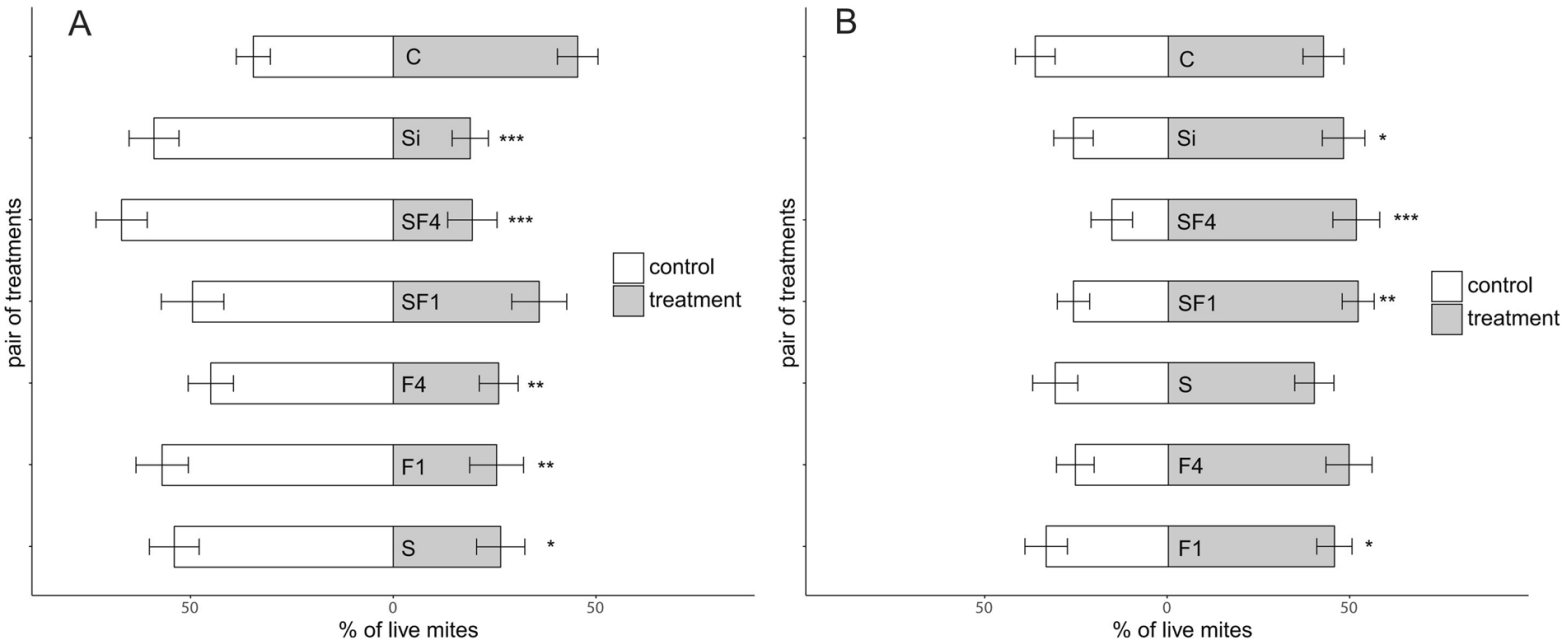
Percentage of live mites (± SEM) in leaf disk two-choice repulsiveness (**a**) (mites initially located on the control leaf) and repellency (**b**) (mites initially located on the treatment leaf) experiments. Asterisk indicates significant differences between treatments compared to the control (C = control; S = soil application; F1 = one foliar application; F4 = four foliar applications; SF1 = soil and one foliar application; SF4 = soil and four foliar applications; Si = silicon application) after 24 h (*p < 0.01; **p < 0.001; ***p < 0.0001; Wilcoxon Signed-Rank test).

In repulsiveness experiments (in which TSSM were initially placed on the control), the dust treatment significantly repelled mites from migration towards the treated disc and/or to settling on the treated disc (Fig. 1a). For one foliar (F1; *N* = 20, *Z* = 2.9, df = 1, *p* = 0.002), four foliar (F4; *N* = 20, *Z* = 3.1, df = 1, *p* = 0.001), soil (S; *N* = 20, *Z* = 2.4, df = 1, *p* = 0.02), soil–four foliar (SF4; *N* = 20, *Z* = 4.1, df = 1, *p* < 0.0001), and silicon (Si; *N* = 20, *Z* = 4.0, df = 1, *p* < 0.0001) applications, the number of mites on the control was significantly higher compared to the treatment after 24 h. Surprisingly, the combination of soil application with one foliar treatment did not impact the mite migration to the treated leaf (SF1; *N* = 20, *Z* = 1.3, df = 1, *p* = 0.19). With regard to mortality, there was a significant difference between mites located on the treatment and control, and specifically the number of dead individuals was higher in the control than in treated groups for four foliar (F4; *N* = 20, *Z* = 3.4, df = 1, *p* = 0.001) and silicon (Si; *N* = 20, *Z* = 2.4, df = 1, *p* = 0.03) treatments (Table 2). Some mites may have migrated back to the control after being exposed to the treatment. Interestingly, the soil–foliar applications did not show significant mortality compare to the control.

**Table 2:** Mean percentage mortality (± SEM) of mites in two-choice bioassays and at different treatments after 24 h.

Pair of treatments	Mortality (± SEM) %					
	Repellency^a^	*Z*	*p*^§^	Repulsiveness^b^	*Z*	*p*^§^
Control	0.0 (± 0.0)	–	–	4.0 (± 1.7)	–	–
Control	1.0 (± 0.1)	− 1	1	0.5 (± 0.5)	1.81	0.1
Control	1.0 (± 0.7)	–	–	4.0 (± 2.4)	–	–
Foliar (F1)	11.0 (± 3.9)	− 2.58	**0.01**	1.5 (± 0.8)	1.93	0.06
Control	1.0 (± 0.7)	–	**–**	6.5 (± 1.5)	**–**	**–**
Foliar (F4)	8.5 (± 2.4)	− 3.56	**< 0.001**	0.5 (± 0.5)	3.41	**0.001**
Control	1.0 (± 0.7)	–	**–**	4.0 (± 1.7)	**–**	**–**
Soil (S)	10.5 (± 2.7)	− 3.13	**0.002**	1.0 (± 0.7)	1.59	0.18
Control	0.5 (± 0.2)			2.0 (± 1.1)	**–**	**–**
Soil–foliar (SF1)	14.0 (± 3.0)	− 3.73	**< 0.001**	2.0 (± 1.2)	0	1
Control	0.0 (± 0.0)	–	**–**	3.5 (± 1.8)	**–**	**–**
Soil–foliar (SF4)	16.5 (± 4.7)	− 3.78	**< 0.001**	1.0 (± 0.7)	0.96	0.37
Control	0.0 (± 0.0)	–	**–**	4.5 (± 1.5)	–	–
Silicon	10.5 (± 2.7)	− 4.03	** < 0.001**	0.5 (± 0.5)	2.37	**0.03**

^§^Bold values indicate significant differences of means to the control. Wilcoxon–Mann–Whitney test.

^a^Mites initially located on the treatment leaf.

^b^Mites initially located on the control leaf.

In repellency experiments (in which mites were initially located on the treatment), there were no significant differences in the distribution of TSSM between four foliar dust (F4) and soil application (S) treatments and control, and after 24 h the mites were equally distributed between treatments (Fig. 1b). However, mites tended to stay on leaves treated with one foliar application (F1; *N* = 20, *Z* = − 2.6, df = 1, *p* = 0.007), the two combinations of soil and one foliar (SF1; *N* = 20, *Z* = − 3.3, df = 1, *p* < 0.001) and soil and four foliar (SF4; *N* = 20, *Z* = − 3.9, df = 1, *p* < 0.001), as well as silicon (*N* = 20, *Z* = − 2.4, df = 1, *p* = 0.01). Migration has still occurred between leaf discs indicating some degree of repellency exerted by the dust treatment via foliar and soil applications; however, the combination of the two treatments was not effective in repelling mites. A significant acaricidal effect increased over time for all treatments (soil, foliar, soil–foliar, and silicon), with the only exception of SF1 at 24 h. Mites may be susceptible to rock dust action when exposed by contact and/or by indirect action through the feeding performance and digestion efficiency of the plant material treated with dust. In both two-choice experiments, mites were equally distributed between two control leaf disks not showing a significant preference (repellency, *N* = 20, *Z* = − 1.3, df = 1, *p* = 0.19; repulsiveness, *N* = 20, *Z* = 1.03, df = 1, *p* = 0.31).

In no-choice experiments, there were statistically significant differences among rock dust treatments, which increased (acute) mite mortality versus the control (F_6,247_ = 19.2, *p* < 0.0001), and (chronic) mortality over time (F_1,247_ = 61.9, *p* < 0.0001) (Table 3). The combination of one dust foliar treatment and dust application as soil amendment (SF1) was not significant compared to the control in inducing mortality 24 h after mite introduction, while four dust foliar treatments and dust application as soil amendment (SF4) and the separated application via foliar (F1 and F4) or via soil (S) exerted strong acaricidal activity, similarly observed for the silicon treatments (Table 3). However, after 48 h all the treatments induced significant mortality affecting between 40–60% of the overall mite population versus the control.

**Table 3:** Mean percentage mortality (± SEM) of mites at different time point and different treatments in no-choice bioassays.

Treatment	ID	Mortality (± SEM) %					
		24 h	*z*	*p*^§^	48 h	*z*	*p*^§^
Control	C	7.5 (± 1.7)	–	–	15.0 (± 2.4)	–	–
Foliar 1	F1	35.0 (± 4.1)	6.031	** < 0.001**	55.0 (± 3.8)	7.231	** < 0.001**
Foliar 4	F4	24.5 (± 2.8)	3.728	**0.005**	44.0 (± 3.6)	5.242	** < 0.001**
Soil	S	31.5 (± 4.9)	5.264	** < 0.001**	45.5 (± 6.1)	5.513	** < 0.001**
Soil–foliar 1	SF1	20.5 (± 2.4)	2.851	0.07	40.5 (± 2.8)	4.609	** < 0.001**
Soil–foliar 4	SF4	22.5 (± 3.1)	3.290	**0.02**	33.5 (± 2.9)	3.344	**0.02**
Silicon	Si	33.0 (± 3.5)	5.592	** < 0.001**	44.5 (± 5.1)	5.333	** < 0.001**

^§^Bold values indicate significant differences between treatment and control. F-test (sum of squares).

The elemental analysis performed on tomato leaves from different treatments reported higher concentrations of silicon (Table 4) in foliar treatments (F_6,14_ = 17.67, *p* < 0.0001); however, the application of rock dust or a diatomaceous earth-based product as soil amendment did not affect the presence of silicon in plant tissues.

**Table 4:** Effects of different treatments on silicon contents (ppm ± SEM) in tomato leaves.

Treatment	Silicon		
	ppm	*t*	*p*^§^
Control	1,350.0 ± 219.0	–	–
Foliar 1	5,063.3 ± 297.8	5.7	** < 0.001**
Foliar 4	4,683.3 ± 922.5	5.1	** < 0.001**
Soil	920.0 ± 110.2	0.7	0.5
Soil–foliar 1	4,043.3 ± 376.5	4.1	**0.001**
Soil–foliar 4	4,800.0 ± 599.1	5.3	** < 0.001**
Silicon	913.3 ± 24.0	0.7	0.5

^§^Bold values indicate significant differences of means to the control.

**Figure 2:**
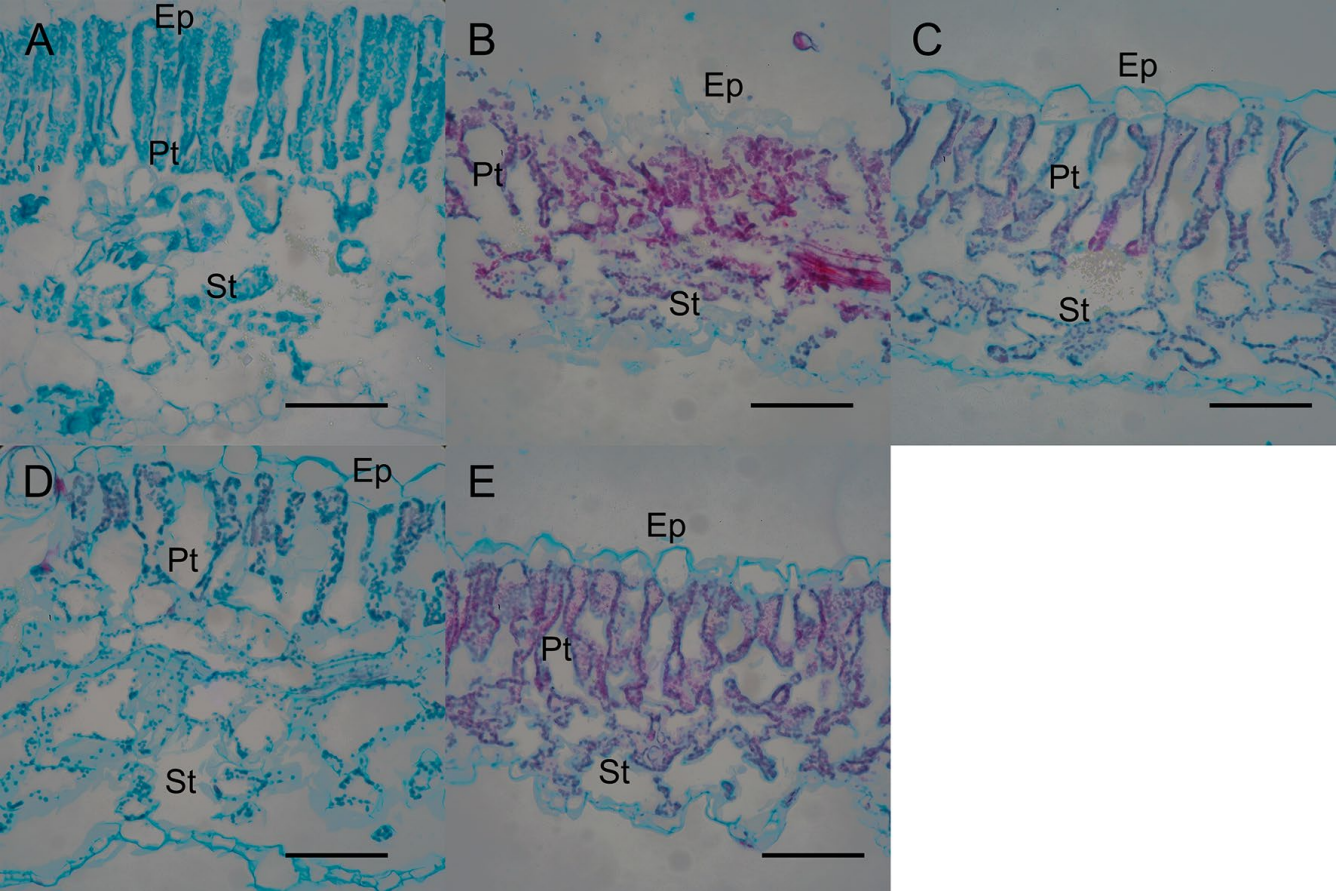
Safranin-O/Fast Green staining of leaf blade cross section of tomato leaves exposed to different treatments (A = control; B = four foliar applications; C = silicon application; D = soil application; E = soil and four foliar applications). Ep = epidermis; Pt = palisade tissue; St = spongy tissue. All scale bars are = 100 μm.

Histological analyses of tomato leaves indicated differential interactions with one of the staining agents (i.e., safranin-O) that revealed a possible morphological variation in the leaf structure (Fig. 2). A red/pink coloration inside the palisade mesophyll cells was observed in leaf tissues that have been exposed to rock dust or diatomaceous earth-based product revealing presence of lignified tissue^26^, while the control leaves reported a minimum or almost absent red/pink coloration. From a qualitative analysis, the epicuticle in leaf tissue treated with rock dust appeared to be thicker compared to control leaf tissue and leaf tissue treated with diatomaceous earth-based product. Moreover, differences were noticed among treatments in term of palisade mesophyll cell density and spongy tissue distribution.

## Discussion

The treatment of granite dust through foliar and soil application exerted repellent and acaricidal activity towards two-spotted spider mites, reporting a dust/herbivore interaction either when the product was applied in the growth medium, or as foliar treatment alone, or as foliar treatment in combination with the soil application. Chemical analyses on the elemental composition of the granite dusts reported a high level of silica and other oxide (the chemical composition is protected intellectual property of Heritage Memorials Ltd.), which suggests a correlation to silicon in term of pest control activity as previously reported for other Si-based products (e.g., diatomaceous earth) that exert a similar effect and mode of action in protecting plants against abiotic and biotic factors^6,7,16,27,28^. The elemental analysis on tomato leaf material confirmed the accumulation of silicon on plant tissues when rock dust is applied on the leaves, possibly impacting the ability of mites to feed. Interestingly, when rock dust was applied as soil amendment the accumulation of silicon on the leaves was not significantly different from the control. Although silicon accumulation process has been previously described^29^, as dicotyledons tomato plants are usually poorer in silicon uptake^10,30^. Si accumulation differs between plant species and it has been attributed to the ability of the roots to take up Si, as well as the different uptake mechanism used^31^. Compared to other plant species such as rice^32^, wheat^33^, or grapevines^34,35^, tomato plants accumulate low level of Si from an external source, resulting in less susceptibility to the beneficial effects of Si exposure in increasing resistant to herbivory attack^30,36^.

The action of silicon in promoting plant resistance has not been well characterized, but evidence for *ex planta* and *in planta* processes are given indicating multiple combined effects rather than one single effect^10^, and silicon has been proposed to act either directly or indirectly on insect herbivores^37^. Several studies have been performed to investigate the effects of Si as a nutrient capable of providing some measures of defense in plants against herbivores and pathogens^14,33,38–42^. It is generally recognized that silicon is able to affect folivorous, phloem-feeding, and some xylem-feeding pests, and action is not only linked to leaf mechanical properties^41,42^, but also to indirect induced chemical defenses putatively mediated by soluble Si^7,8,37,43^. The morphological changes observed at the tissue level after exposing plants to a silicon source may be linked to an increase in lignified cells^44^ and to epidermal thickening (Fig. 2), affecting the herbivore feeding. Two-spotted spider mites feed on cell sap within the leaf mesophyll^45^; in plants exposed to a silicon source, tissue was colored red from the safranin-O stain, indicating an increasing of the lignification process in the palisade and spongy mesophyll tissues. Other than inducing production of lignified cells, it has been suggested that silicon may be incorporated into cell walls as silicon-aromatic ring associations between lignin and carbohydrate in leaves of some plant species^46^, playing a role in reducing pest damage and spread of infection^43^. As previously observed, silicon is able to augment stress-induce lignin accumulation in plants, initiating defense pathways faster^47,48^.

Two-spotted spider mites stayed on the control leaf (repulsiveness) or distributed evenly between treatments (repellency) when presented with a granite dust—treated leaf (Fig. 1). Because of the resulting abrasiveness of leaf surface, foliar application might have provided a mechanical barrier and an increased physical resistance to the penetration by feeding mites. Other than acting as a physical deterrent, granite dust has shown significant acaricidal properties after contact causing between 8–16% (in two-choice experiments) and 20–45% (in no-choice experiments) mortality rate. Particularly, the survival rate significantly decreased over time when mites do not have a choice (Table 3). Acaricidal activity was already reported for other similar dust products (i.e. diatomaceous earth and high-silica content materials) mainly linked to sorptive properties of the particles and to a desiccation process^49^.

In no-choice experiments the combined treatment of foliar and soil application exerted an equal effect in reducing the survival rate of the mite population, with results not statistically different from the other two separated treatments (soil and foliar) and silicon after 48 h (Table 3). In repulsiveness experiments, mortality rate was not significantly different between control and soil–foliar treated leaves (SF1 and SF4) as well as one foliar treatment (F1); however, in the four foliar application (F4) a significant mortality was recorded for the mites located on the control leaf (Table 2). A possible explanation may consider the potential greater repellent action imposed by the two soil–foliar treatments and the one foliar application, inducing mites to avoid treated leaves, thus reducing the exposure time to the dust, while in the case of four foliar application mites, that have been exposed to the rock dust, have died after returning to the control leaf (Fig. 1a). In two-choice experiments, mites had 24 h in order to make a choice between the two treatments and settling in, and during this time they may have being exposed to the treatment and being negatively affected by the dust via contact or during the feeding process. It has been previously observed that introduction of granite dust as a soil amendment has an effect on cell wall reinforcement in various test plants^44,47,50^. This effect may have impacted feeding, causing damage on mite mouthparts^27^ and compromising their ability to feed after exposure.

When granite dust was introduced as a soil amendment, the number of mites that settled on the treated leaves was significantly lower than control leaves in repulsiveness two-choice experiments (Fig. 1a), indicating that plants exposed to mineral dust were not suitable as hosts. On the other side, in repellency two-choice tests a migration of mites was observed from treated leaves to control leaves, reporting an equal distribution of mites between treatments after 24 h (Fig. 1b). It has been already reported that the application of mineral products with a source of Si is able to suppress pest infestation; for instance, TSSM was successfully controlled by a potassium silicate formulation applied as a soil amendment^51^. Although the separate application of rock dust as soil amendment or as foliar application exerted a significant repellent action, the combination of the two treatments (SF1 and SF4) did not succeed in repelling the mites from treated leaves.

Numerous studies have shown enhanced resistance of host plants treated with Si to insect herbivores and other arthropods, mostly at two-trophic levels^8,52^. The application of granite dust on tomato for controlling two-spotted spider mites represents another example where a Si-based product is able to control herbivores by providing direct/indirect defence via foliar application and/or in combination as a soil drench. In the future, more studies need to be done in order to elucidate the mechanism of repellent and acaricidal action of the granite dust, involving possible silica uptake content in leaf material after different soil and foliar applications, in relation to the observed pest control and increased level of plant resistance. Moreover, other important crops strongly affected by TSSM (e.g. cucumber, strawberries, eggplants) should be examined under granite dust exposure. Additional investigations are ongoing in order to assess potential impact of granite rock dust on non-target organisms (e.g., pollinators) in order to determine potential toxic effects after topical exposure.

Taken together, our findings demonstrate for the first time that granite rock dust interacts with two-spotted spider mite infestation by modifying pest behavior and via acaricidal action. Moreover, this study contributes to our understanding in the role of mineral products with high Si content, and provides a potentially valuable pest management tool when applied as soil amendment and/or as foliar treatment.

## Methods

### Elemental analysis

Elemental analysis of rock dust samples was performed at the Maxxam Analytics Inc., Bedford, Nova Scotia, Canada. Rock dust samples (N = 11) were collected in different timing between January and November 2018, and were based on grounding material from Stanstead, African Red, Quebec Black stone varieties. Samples were finely grounded as homogeneous material with a diameter range 20–60 μm, and were analyzed for elemental analyses by XRF. Prior analysis, samples were dried at 105 °C to constant weight. XRF data were obtained on fused lithium borate discs (major elements) and pressed powders (trace elements).

Elemental analysis of tomato leaves was performed at Research & Productivity Council Laboratories, Fredericton (NB, Canada). Tomato leaves were sampled from three different plants in each treatment in order to have three replications for each treatment. Leaf samples were washed for removing any rock dust residue and dried in a ventilated oven at 70 °C for 72 h until constant weight, and ground separately. Portions of the samples were prepared by microwave assisted digestion in nitric acid (SOP 4.M26, U.S. EPA Method 26). The resulting solutions were subjected to inductively-coupled plasma mass spectrometry (ICP-MS) analysis for trace elements (SOP 4.M01, U.S. EPA Method 01). Silicon was determined by inductively-plasma emission spectroscopy (ICP-ES) on sodium peroxide fusions of the samples.

### Plants

Tomato seeds (*S. lycopersocum* var. Bonny Best, OSC Seeds, Waterloo, ON, Canada) were grown in the K.C. Irving Centre greenhouse (18 ± 2 °C, 16:8 L:D, 65 ± 5% R.H.) at Acadia University (Wolfville, NS, Canada) in 20-mm-diameter pots containing Pro-Mix potting soil (all-purpose growing mix). After a week, seedlings were transferred in 4 L plastic pots containing Pro-Mix potting soil with or without 10% w/v rock dust depending on the treatment group. Plants were watered daily as needed.

### Treatments

Rock dust powder (RD) (mixture of granite dusts; average particle size 20–60 μm) was provided by Heritage Memorials Ltd. (Windsor, NS, Canada). Plants were exposed to seven treatments: control (C), RD soil (S), RD foliar (one application, F1; four applications, F4), RD soil and foliar (one application, SF1; four applications, SF4), silicon (Si) (Table 5). Each treatment group consisted of five tomato plants. We included silicon treatment applied as soil amendment in order to evaluate a potential connection between the observed acaricidal and repellent activity of rock dust and the absorption of silicon by plants. As a source of silicon, a diatomaceous earth-based product (100% diatomaceous earth, 80% SiO_2_, Insectigone Crawling Insect Killer, Woodstream Canada Corporation, Brampton, ON, Canada) was used. Soil application was prepared by mixing RD at 10% w/w with the Pro-Mix potting soil. Fifteen one-week old tomato seedlings were transferred in pots containing the soil treatment. After a month, we applied the foliar treatment which was prepared by mixing 10% w/v with reverse osmosis (RO) water. RD aqueous solution was then applied on tomato plants (four weeks old) to the point of run-off using a hand-sprayer. Treatment groups exposed to the foliar application were F1, F4, SF1, and SF4. The non-foliar treated groups (C, S, and Si) received RO water in a similar manner (hand-sprayer application). Treatment delivery was completed over a 4-week period. Sampling of leaves for feeding trials started following the fourth foliar treatment. No fertilizer was applied during the treatment and sampling periods.

**Table 5:** List of treatments used in the bioassays.

Treatment	ID	Description
Control	C	Control—no treatment
Rock dust soil	S	Rock dust soil application at 10% w/w
Rock dust foliar	F1	Rock dust foliar application at 10% w/v; foliar treatment applied one time
Rock dust foliar	F4	Rock dust foliar application at 10% w/v; foliar treatment applied weekly (four times in total)
Rock dust soil and foliar	SF1	Rock dust soil (10% w/w) and foliar application (10% w/v); foliar treatment applied one time
Rock dust soil and foliar	SF4	Rock dust soil (10% w/w) and foliar applications (10% w/v); foliar treatment applied weekly (four times in total)
Silicon	Si	Aqueous solution of 2.4–2.5% SiO_2_; 100 ml × 2 soil applications

### Mite colony

TSSM colony was provided by Vineland Research & Innovation Centre (Vineland Station, ON, Canada). Mites were reared on tomato (*Solanum lycopersicum*, var. Moneymaker) foliage in plastic boxes (6L) (37 L × 24 W × 14 H cm) lined with moistened paper towel. Boxes were held in a growth chamber (25 ± 2ºC, 16:8 L:D, 70 ± 5% RH) and tomato foliage was changed every 2–3 days. Adult female two-spotted spider mites were used in the feeding bioassays.

### Feeding bioassays (two-choice)

We used two series of experiments to evaluate the repulsiveness (when mites were placed on plant material free of dust treatment to observe their avoidance to moving onto similar available rock dust treated plant surface) and the repellency (consistent movement of mites from rock dust treated surfaces to similar areas free of rock dust residue) exerted by the dust towards the mites in two-choice leaf disc bioassays. This experimental design exploits the ambulatory dispersal behavior observed in TSSM when unfavourable host/conditions are present^53,54^.

Bioassays were done as a randomized complete block design. Each bioassay had five blocks, and each block had one control and six treatments (10 mites per Petri dish). Bioassays were conducted four times for a total of 200 mites (*N* = 20) per treatment. Every bioassay was conducted using control/treated tomato leaves sampled from plants belonging to the same replication group. Disks (diameter 1.5 cm) from treated and control tomato leaves were cut using a cork borer, and were transferred using forceps with the abaxial surface facing down in plastic Petri dishes (diameter 5 cm) (due to TSSM preference for abaxial leaf surface) lined with moistened filter papers (Whatman no.°1). Each Petri dish was provided with two leaf disks, one control and one treatment placed next to each other. Ten two-spotted spider mites were gently placed on the control leaf disc (for repulsiveness bioassay) or on the treated leaf disk (for the repellency bioassay), and the Petri dishes were closed and sealed with Parafilm. Petri dishes (five pairs of control-treatment) were placed together in a plastic box (17 × 11 × 6 cm) lined with moistened paper towel. Boxes were held in a growth chamber (25 ± 2 °C, 16:8 L:D, 70 ± 5% RH). Repellency and repulsiveness were assessed under a dissecting microscope by determining the number of mites present on control and treated leaf disks 24 h after mite release. Number of dead mites was also recorded by gently probing the mite with a fine brush.

### Feeding bioassays (no-choice)

No-choice experiments were carried out using a similar leaf-disk set-up as described above. Bioassays were done as a randomized complete block design. Each bioassay had five blocks, and each block had one control and six treatments (10 mites per Petri dish). Bioassays were conducted four times for a total of 200 mites (*N* = 20) per treatment. Disks (diameter 1.5 cm) from treated and control tomato leaves were cut using a cork borer, and were transferred using forceps with the abaxial surface facing down in plastic Petri dishes (diameter 5 cm) lined with moistened filter papers (Whatman no. 1). Each Petri dish was provided with one leaf disk (control or treatment). Ten TSSM were gently placed on the leaf disk and Petri dishes were closed and sealed with Parafilm. Petri dishes (control and treatments) were placed together in a plastic box (17 × 11x6 cm) lined with moistened paper towel. Boxes were held in a growth chamber (25 ± 2 °C, 16:8 L:D, 70 ± 5% RH). Mortality was assessed under a dissecting microscope by gently probing the mite with a fine brush 24 and 48 h after mite release. Location of dead and alive mites was also recorded.

### Histochemistry and anatomical assessment

Anatomical studies were conducted using the middle third of the second leaf fully expanded collected from five different plants per treatment, previously fixed in 50% FAA (9:1:1 50% ethanol: Formalin: Glacial Acetic Acid) for 48 h, and subsequently stored in 70% ethanol. Leaf samples were then dehydrated through an ethanol series (80–100%). The ethanol was then replaced with 1:1 100% ethanol: Citrosolve (Advance Chemicals Ltd., Saskatoon, SK, Canada), and then 100% Citrosolve. Paraffin chips (Sigma-Aldrich, Saint Louis, MO, USA) were added to the leaf samples in Citrosolve and left at 60 °C for 72 h. After three changes of fresh wax, leaf tissues were embedded in paraffin wax using the Leica EG1160 embedding system (Leica Biosystems, Canada). Transverse sections (10 μm thickness) were obtained using an AO820 Rotary Microtome, transferred to glass slides. Microscope slides were prepared for staining by placing them in a solution of Histo-Clear (National Diagnostics, Atlanta, GA, USA) to deparaffinize them, and rehydrated through an ethanol series (100%, 95%, 80%, and 70%) before being stained overnight in a Safranin stain solution^55^. The following morning, slides were taken through a series of washes and counterstained with Fast Green FCF before being coverslipped with Permount mounting media. Tissue sections were observed with a Nikon Eclipse 50i compound microscope and photographed with a Nikon D700 DSLR camera at 40x. TIF images were then imported into Adobe Lightroom Classic and edited for white balance and clarity.

### Statistical analysis

Statistical analyses were conducted with RStudio Version 01.1453^56^. Data with non-normal distribution were subjected to non-parametric tests (i.e., Kruskal–Wallis). Mortality from no-choice feeding experiments and differences in silicon concentration between treatments were analyzed with a linear mixed-effect model (lmer). We compared the linear mixed effects model with F-test (sum of squares) with Kenward-Roger approximation^57^ to determine the best fit and we performed a post-hoc least-squares means corrected for multiple testing (Tukey method; emmeans package) on the model to determine differences between groups. In order to analyze mortality over time, the model was structured as repeated measures, with time included as fixed effect, and Petri dish arena as a random block effect. For two-choice feeding experiments (repulsiveness and repellency), results were analyzed by Wilcoxon Signed-Rank Test (α = 0.05). Differences were considered significant at *p* ≤ 0.05.
